# A canine identity crisis: Genetic breed heritage testing of shelter dogs

**DOI:** 10.1371/journal.pone.0202633

**Published:** 2018-08-23

**Authors:** Lisa M. Gunter, Rebecca T. Barber, Clive D. L. Wynne

**Affiliations:** 1 Department of Psychology, Arizona State University, Tempe, Arizona, United States of America; 2 Mary Lou Fulton Teachers College, Arizona State University, Tempe, Arizona, United States of America; University of Missouri Columbia, UNITED STATES

## Abstract

Previous research in animal shelters has determined the breeds of dogs living in shelters by their visual appearance; however the genetic breed testing of such dogs is seldom conducted, and few studies have compared the breed labels assigned by shelter staff to the results of this testing. In the largest sampling of shelter dogs’ breed identities to-date, 459 dogs at Arizona Animal Welfare League & SPCA (AAWL) in Phoenix, Arizona, and 460 dogs at San Diego Humane Society & SPCA (SDHS) in San Diego, California, were genetically tested using a commercially available product to determine their breed heritage. In our sample, genetic analyses identified 125 distinct breeds with 91 breeds present at both shelters, and 4.9% of the dogs identified as purebreds. The three most common breed signatures, in order of prevalence, American Staffordshire Terrier, Chihuahua, and Poodle, accounted for 42.5% or all breed identifications at the great grandparent level. During their stay at the shelter, dogs with pit bull-type ancestries waited longer to be adopted than other dogs. When we compared shelter breed assignment as determined by visual appearance to that of genetic testing, staff at SDHS was able to successfully match at least one breed in the genetic heritage of 67.7% of dogs tested; however their agreement fell to 10.4% when asked to identify more than one breed. Lastly, we found that as the number of pit bull-type relatives in a dog’s heritage increased, so did the shelter’s ability to match the results of DNA analysis. In total when we consider the complexity of shelter dog breed heritage and the failure to identify multiple breeds based on visual identification coupled with our inability to predict how these breeds then interact within an individual dog, we believe that focusing resources on communicating the physical and behavioral characteristics of shelter dogs would best support adoption efforts.

## Introduction

Every year nearly 4 million dogs enter an animal sheltering organization in the United States either surrendered by their owners, as strays, returned after adoption, or confiscated as part of cruelty and criminal cases [1]. Stray dogs likely compose 53–77% of shelter canine populations in the US, varying by geographic area and type of shelter [2–4]. Dogs that enter animal shelters as strays are without an owner, and their history and breed heritage is typically unknown. Prior research has shown that a majority of dogs arriving into shelters appears to be mixed breed [5,6].

The American Pet Products Association (APPA) estimates from survey data that 78 million dogs live in American households [7]. These were most commonly obtained from a family member or friend, an adoption organization, or a breeder [8]. The largest breed registry of purebred dogs in the United States, the American Kennel Club, presently recognizes 189 individual dog breeds (http://www.akc.org). In the late 1990s, purebred dogs were thought to have comprised approximately 50% of dogs in homes [9]. Today, as the number of dogs registered to the AKC declines [10], it is possible that the percentage of purebred dogs in homes may also be waning, but no reliable data are available.

When Hoffman, Harrison, Wolff, and Westgarth [11] asked animal shelter workers what criteria they used to determine breed assignment of dogs of unknown heritage, physical appearance was indicated as the primary means of identification, with characteristics such as the dog’s size, weight, musculature, legs, tail and coat often mentioned. Considering the abundant diversity in modern dog breeds [12], morphological features may be one means to differentiate purebred dogs.

Notwithstanding its prevalence in animal sheltering, however, visual identification of breeds based on morphology has consistently failed to describe the breed heritage of mixed breed dogs when compared to DNA analysis. Voith, Ingram, Mitsouras, and Irizarry [13] reported that shelter staff matched one breed within the dog’s heritage in 7 out of 20 subjects; and in those cases, that breed often represented only one-eighth of the dogs’ total breed make-up. When dog professionals were provided with videos for identification of these same 20 mixed breed dogs, on average fewer than 30% of participants were able to identify one breed in the dog’s DNA analysis [14]. Furthermore inter-rater reliability was low; and even when agreement about the predominant breed was greater than 50%, the breeds agreed upon for three of the seven dogs were not found in their genetic analyses.

These issues are particularly acute in the assessment of dogs belonging to the group commonly known as “pit bulls.” Beginning in the 1980s, pit bulls have been characterized as a dangerous breed [15], particularly from their history of association with dog-fighting and implication in dog-bite injuries and deaths [16–18]. Consequently, breed-specific legislation was enacted across the United States to address this risk with local ordinances ranging from prohibiting ownership [19], to confinement restrictions and muzzling [20], to mandatory sterilization [21]. Thus far, limited empirical data has been published on the effect of these legislations on improved public safety; however breed bans in Spain [22], the Netherlands [23], Canada [24], and Italy [25] have failed to decrease bite incidents and a recent study from Ireland found no differences between restricted and non-restricted breeds in the severity of bites inflicted or the likelihood that the bite would need greater medical attention [26].

One complication in understanding the risk posed by pit bulls is identifying these dogs. The aforementioned US jurisdictions that ban these dogs use the preponderance of physical characteristics associated with the breeds of American Pit Bull Terrier, American Staffordshire Terrier or Staffordshire Bull Terrier as their means of positive identification. However breed characterization based on morphology has been found to be inconsistent among individual assessors and an unreliable means of identification compared to DNA analysis. Olson et al. [27] reported that 50% of dogs assessed as pit bulls at a Florida shelter lacked the signatures of the breeds associated with that label. Additionally, recent research has indicated that the label of “pit bull,” independent of the dog’s visible characteristics, can influence perceptions of a dog’s attractiveness to potential adopters, as well as the dogs’ length of stay in the shelter and adoption success [28].

Advances in canine genomics allowed the advent of commercial genetic breed testing, making possible the identification of component breeds within mixed breed dogs. In 2005, researchers reported the first genomic draft sequencing of the domestic dog along with mapping of two-and-a-half million single nucleotide polymorphisms (SNPs) [29]. Koskinen [30], Irion et al. [31], and Parker et al. [32] successfully assigned purebred dogs to their distinct breeds through the genotyping of varying numbers of allelic microsatellite markers. Boyko et al. [33] used DNA samples from 915 dogs across 80 dog breeds (as well as a number of wild canids) to identify a relatively small number of genes of large effect that are responsible for physical traits such as body size, coat length, ear type, and snout length (see also Vaysse et al. [34]). These developments are particularly noteworthy considering the relatively recent origins of dog breeds themselves, and allow for group classifications in the canine population based on genetic variation, and not solely their roles in society or their physical appearances [35].

The present paper has two main aims. First, to report the breed heritage of a large sample of mixed breed shelter dogs based on genomic breed testing. We identify the breed signatures and the number of breeds detected in our sample, the amount a single breed typically contributed to a dog’s breed heritage, the proportion of purebred dogs identified at the sheltering organizations and the impact of breed on length of stay. Second, to assess agreement of visual breed identification by shelter staff at one of these locations by comparing the primary and secondary breeds indicated by staff and those identified by DNA analysis.

## Methods

### Subjects

From December 2014-August 2015, all dogs newly admitted to the Arizona Animal Welfare League & Society for the Prevention of Cruelty to Animals (AAWL: Phoenix, AZ, USA) were enrolled in the study; and from April 2015-April 2016, dogs admitted to the San Diego Humane Society and Society for the Prevention of Cruelty to Animals (SDHS: San Diego, AZ, USA) were enrolled. AAWL and SDHS are limited-admission private animal shelters. At AAWL, nearly half of all dogs (47%) were transferred to the shelter from other welfare organizations. 38% of dogs were brought to AAWL by their owners (including returns) with only 15% arriving as strays. At SDHS, over forty percent of dogs (41.5) arrived as owner surrenders or returns with 31.4% as strays and only a small proportion (11.4%) transferred from nearby shelters. We collected records via software programs, PetPoint (Oakville, ON, CAN) at AAWL, and Shelter Buddy (Englewood, CO, USA) at SDHS, and we used the dogs’ intake dates, outcome dates and types, and primary and secondary breeds in our analysis.

### DNA breed testing

Buccal cells were collected from dogs’ cheeks and gums via cytobrush kits provided by Wisdom Panel Canine DNA Tests (Mars Veterinary, Portland OR). These samples were allowed to dry and then placed into individual protective sleeves. Sleeves were collected into overnight courier envelopes in batches and shipped to GeneSeek Laboratory (Lincoln, NE) twice weekly for processing. Each kit number was activated online at the Mars Veterinary Wisdom Panel Business Portal (http://business.wisdompanel.com) prior to shipment. Once a kit was activated with the swab’s sample ID and identifying information about the dog (i.e., name or shelter ID number), the laboratory then uploaded results to the Kit Status Checker area of the portal when analysis was completed.

With the Mars Wisdom Panel 2.0 DogTrax product, DNA is extracted from the buccal cells and typed at 321 single nucleotide polymorphisms (SNPs) across the canine genome using PCR amplification and base-specific cleavage. The Sequenom platform (Sequenom, San Diego, CA) was used for SNP genotype detection by matrix-assisted laser desorption/ionization time of flight mass spectrometry (MALDI TOF-MS) [36]. Bayesian generative modeling utilizing a Mars Veterinary-proprietary Markov Chain Monte Carlo sampling process was used to translate genotype information to breed matches and develop a best-fit family tree model.

The Mars DNA database from which these breed signatures were identified was developed through the genotyping of over 13,000 purebred dogs. Mars Veterinary sampled 246 mixed breed dogs of known heritage to establish accuracy measurements for the Wisdom Panel 2.0 DogTrax product. The genetic breed test was found to have an overall positive predictive value greater than 90% at standard confidence levels for reporting of breeds within a dog’s heritage. This accuracy was not dependent on number of breeds identified (Mars Veterinary, personal communication, January 4, 2018).

The Wisdom Panel DogTrax report includes breed signatures from three generations of ancestors from 209 breeds and varieties (S1 Table) with the use of “mixed breed” for relatives in which no distinct purebred signature could be identified. Any ancestry contribution under approximately 12.5% is not reported. For this study, the two most predominant breeds were designated on the Wisdom Panel report analogous to the primary and secondary breeds reported on shelter kennel cards.

### Shelter breed assignment

At SDHS, the dogs’ primary and secondary breeds as determined by shelter staff were collected prior to receiving the results of the dogs’ DNA tests. Breed assignment followed the shelter’s current protocol based on visual identification. To assist in the visual breed identification of these dogs, shelter staff were provided with American Kennel Club Breed Identification Guides [37] and encouraged to use breed labels that were recognized breeds. A minimum of a primary breed was provided, but staff could also indicate a secondary breed or mix.

This study was exempted from review by the Arizona State University Institutional Review Board.

### Statistical analysis

Breeds reported from the Wisdom Panel DogTrax report were counted in two different ways. First, we calculated the total number of individually identifiable breeds and varieties found in the dogs analyzed in this study. Breed varieties include dogs of the same breed for which distinct subpopulations have been identified and assigned genetic signatures, such as by different countries of origin (e.g., the United States and United Kingdom), use (e.g., field and show), size (e.g., miniature and toy), and coat (e.g., longhaired and shorthaired). For the second analysis, varieties were reduced to their single breed population (such as Beagle or Dachshund). Where a single breed signature could not be conclusively identified at great-grandparent level, we applied the label “mixed”.

Shelter breed identification data was standardized prior to analysis to remove data entry inconsistencies, spelling errors and to combine certain breed variations into single breed groupings (as above with dogs of the same breed that have different size and coat varieties). In addition, 10 dogs were excluded due to having been identified by a generic label (four “Shepherds”, two “Terriers”, and two “Spaniels”), or in cases where the Wisdom Panel DogTrax reports do not detect that breed (the only case was one Korean Mastiff), or where the breed is a recent hybrid (the only case was a “Labradoodle”). An additional 50 dogs were excluded because no visual identifications were recorded by staff. Length of stay (LOS) was defined as the number of days housed at the shelter from day of intake to day of outcome.

## Results

### Genetic breed markers

DNA analysis was performed and results returned for a total of 919 dogs, 459 at AAWL and 460 at SDHS. A total of 186 identifiable breeds and varieties were identified: 168 at AAWL and 166 at SDHS. Twenty of these signatures were exclusively found at AAWL, and 18 at SDHS. When signatures of different varieties of the same breed were combined into a single breed, 125 breeds were identified within the total sample, and 91 of those breeds (72.8%) were present at both AAWL and SDHS.

**Fig 1:**
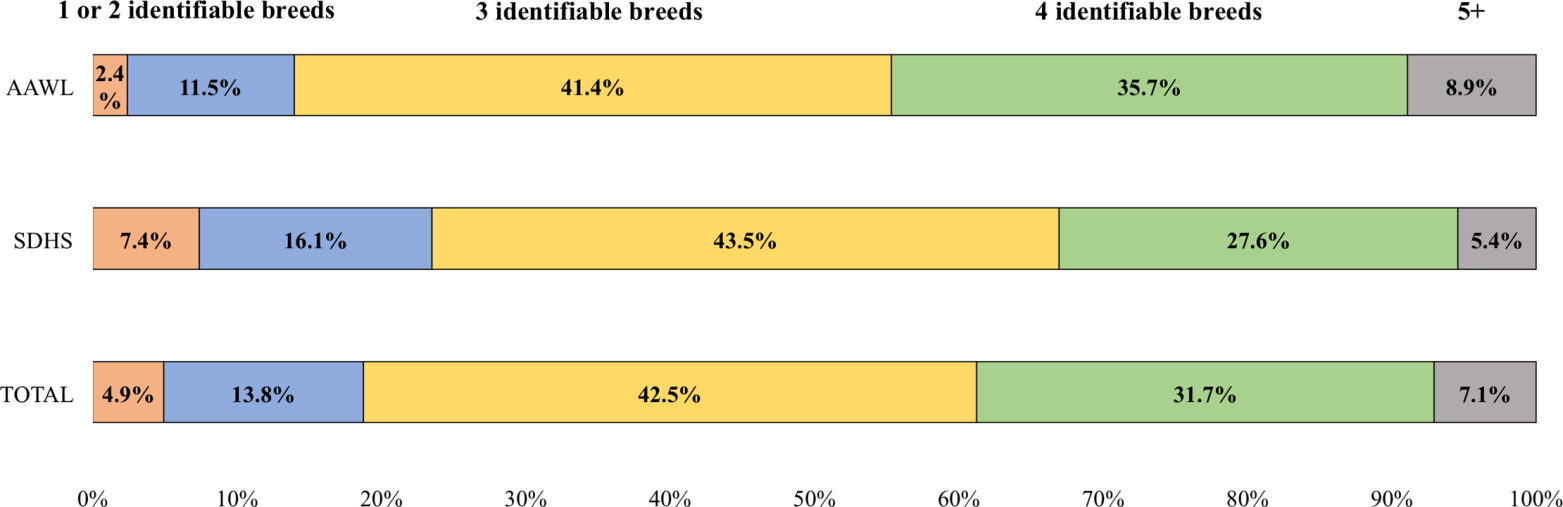
Proportion of dogs by number of identifiable breeds within its breed heritage.

At the great-grandparent (GGP) level, 87.8% of the dogs had at least one relative who was identified as “mixed” (unidentifiable) breed. 4.9% of the 919 dogs (2.4% at AAWL, 7.4% at SDHS) were identified as being purebred dogs, with the most commonly identified being Labrador Retriever (5 individuals), American Staffordshire Terrier (5), and Yorkshire Terrier (5). In total, the 45 purebred dogs represented 22 breeds (Table 1). An additional 12 dogs (1.3%) were identified as having had two purebred parents of different breeds (Table 2). Only 11.6% could be identified with one specific breed and no other purebred GGPs (the combination typically labeled a breed “mix”). Thus, a total of 18.7% of dogs could be identified by a single breed, two specific breeds, or one breed plus “mixed.” The remaining dogs had at least two identifiable breeds plus “mixed” in their three-generational breed heritage. While most dogs were of a multiple breed heritage, 44.5% of the dogs (AAWL: 42.7%, SDHS: 46.3%) were found to be at least 50% of one specific breed (Fig 1).

**Table 1:** Number and breeds of purebred dogs at Arizona Animal Welfare League and San Diego Humane Society.

Breed	AAWL	SDHS	Total
American Bulldog		1	1
American Staffordshire Terrier	1	4	5
Border Collie		1	1
Boston Terrier		1	1
Boxer	3	1	4
Bulldog		1	1
Chihuahua	1	2	3
Cocker Spaniel		1	1
Doberman Pinscher		1	1
German Shepherd Dog		2	2
Golden Retriever		1	1
Labrador Retriever		5	5
Miniature Pinscher		1	1
Pomeranian	1	1	2
Pug		1	1
Rottweiler	1		1
Russell Terrier		1	1
Saint Bernard	1		1
Schnauzer	1	2	3
Shih Tzu		3	3
Siberian Husky		1	1
Yorkshire Terrier	2	3	5
Total purebreds	11	34	45

**Table 2:** Dogs at Arizona Animal Welfare League and San Diego Humane Society with two purebred parents of different breeds.

Breeds	AAWL	SDHS	Total
Chihuahua, Rat Terrier	1	2	3
Chihuahua, Russell Terrier	1		1
Dachshund, Yorkshire Terrier	1		1
Great Dane, Saint Bernard	1		1
Labrador Retriever, Chihuahua		1	1
Pekingese, Rat Terrier	1		1
Poodle, Chihuahua	1		1
Poodle, Schnauzer	1		1
Yorkshire Terrier, Maltese		1	1
Yorkshire Terrier, Poodle	1		1
Total purebred crosses	8	4	12

The most common breeds identified at both shelters were similar. 24% of dogs at AAWL and 27.8% of dogs at SDHS had at least one American Staffordshire Terrier GGP. Broadening the analysis to include all the breeds typically classed as “pit bull-type” (dogs with at least one GGP from American Staffordshire Terrier, American Bulldog, Bull Terrier, and Staffordshire Bull Terriers), these dogs accounted for 26.6% of intakes at AAWL and 30.7% of intakes at SDHS. The second most common breed signature at both shelters was Chihuahua, with 24% of AAWL dogs and 17.8% of SDHS having at least one GGP of Chihuahua heritage. Poodle was the third most common breed signature, appearing in 15.3% of the AAWL dogs and 14.6% of SDHS dogs. Although the order of prevalence varied, Boxer, German Shepherd Dog, Labrador Retriever, Cocker Spaniel and Dachshund were in the 10 most commonly identified breeds for both shelters, although in most cases the average concentrations for these breeds dropped closer to two GGP (Tables 3 and 4).

**Table 3:** Most commonly observed breed signatures at Arizona Animal Welfare League.

Breed	Count	% of shelter sample	Avg. concentration (%)
American Staffordshire Terrier	110	24.0	37.4
Chihuahua	110	24.0	38.0
Poodle	70	15.3	32.9
Boxer	51	11.1	34.6
German Shepherd Dog	39	8.5	24.7
Labrador Retriever	37	8.1	20.9
Cocker Spaniel	31	6.8	23.4
Australian Cattle Dog	30	6.5	25.8
Dachshund	27	5.9	20.8
Shih Tzu	25	5.4	29.5
Mixed (beyond three generations)	419	91.3	37.3

**Table 4:** Most commonly observed breed signatures at San Diego Humane Society.

Breed	Count	% of shelter sample	Avg. concentration (%)
American Staffordshire Terrier	128	27.8	45.5
Chihuahua	82	17.8	39.6
Poodle	67	14.6	30.6
German Shepherd Dog	55	12.0	30.0
Labrador Retriever	39	8.5	35.9
Boxer	38	8.3	28.6
Cocker Spaniel	34	7.4	25.0
Yorkshire Terrier	30	6.5	41.3
Dachshund	25	5.4	22.0
Maltese	22	4.8	25.6
Mixed (beyond three generations)	388	84.3	37.4

Although pit bull-type dogs were the most common breed signatures identified at the GGP level at both shelters, the average concentration of pit bull ancestry (the percentage of that breed in an individual dog’s heritage) was low to moderate. On average, dogs at AAWL identified as having a pit bull-type breed in their heritage had a concentration of 38.5% (approximately three GGP out of eight), and SDHS dogs had a concentration of 48.4% (nearly 4 GGP out of eight).

As observed with pit bull-type dogs, the average concentration of Chihuahua GGP in dogs with any Chihuahua breed signatures was relatively low. Out of a combined total at both shelters of 193 dog with Chihuahua signatures, only three were purebred. Both shelters saw average Chihuahua breed concentrations of 38–39%, indicating just over three GGP out of eight in an individual dog’s heritage.

### Length of stay

The length of stay (LOS) at the two shelters was comparable, with dogs at AAWL kenneled an average of 23.6 days awaiting adoption while SDHS dogs’ average LOS was 25.8 days. There was a noticeable difference, however, between dogs with signatures of pit bull-type breeds when compared to dogs without. The difference was similar at both shelters, but more pronounced at SDHS.

Dogs with pit bull-type ancestry as identified by DNA analysis at both shelters had a mean length of stay nearly twice as long as non-pit bull-type breeds. On average, dogs with no DNA contribution from pit bull-type breeds stayed in the shelter for 19.7 days as compared to 37.5 days for dogs with at least one pit bull GGP (Table 5). When disaggregated by shelter, however, there was a difference in magnitude. At SDHS, a significant independent samples t-test was found for the 23.2-day difference in overall length of stay between pit bull-type breeds and other dogs. In contrast, that difference was only 12.0 days at AAWL, which, while still statistically significant, is half the difference found at SDHS. It should be noted that in both cases Levene’s Test for Equality of Variances failed, resulting in substantial reductions in the degrees of freedom to adjust for the variance differences. Table 6 provides details of these test statistics.

**Table 5:** Length of stay (LOS) in days for pit bull-type dogs and all other breeds at Arizona Animal Welfare League and San Diego Humane Society.

Breed group	Count	Minimum LOS	Maximum LOS	M	SD
Pit bull-type dogs	244	1	333	37.5	49.41
AAWL	117	1	333	32.5	49.05
SDHS All other breeds AAWL SDHS	127 626 328 298	1 1 1 1	249 260 233 260	42.0 19.7 20.5 18.9	49.49 25.02 21.57 28.35

**Table 6:** Significance tests for pit bull-type dogs’ and all other breeds’ length of stay (LOS) at Arizona Animal Welfare League and San Diego Humane Society.

	t-value	df	*M* difference	*95% CI*	
				LL	UL
Overall	5.351**	293	17.751	11.222	24.280
AAWL	2.560*	132	12.001	2.727	21.274
SDHS	4.946**	162	23.188	13.930	32.446

*Note*. CI = confidence interval; LL = lower limit, UL = upper limit.

* p < .05.

** p < .001.

Percentage of pit bull heritage correlated positively with length of stay at SDHS (*r* (127) = .373, *p* < .001), but not at AAWL (*r* (117) = .039, *p* = .673). The percentage of mixed heritage (where a specific breed could not be identified at the great-grandparent level) was not significantly correlated with length of stay at AAWL. However, at SDHS length of stay was significantly negatively correlated with the percentage of mixed breed identified (*r* (360) = -.152, *p* = .004).

While the total number of identifiable breed signatures contributing to a dog was not significantly correlated with its length of stay at either shelter, there was a small but significant positive relationship at SDHS between length of stay and a dog having at least 50% of its DNA from a single breed signature (*r* (425) = .112, *p* = .02). Further investigation of this result found that the significant correlation was only for dogs of pit bull-type breed heritage, (*r* (127) = .220, *p* = .013).

### Staff identification of breed

Visual identification of the dog’s primary breed by shelter staff matched the most prevalent breed identified by the Mars Wisdom Panel DogTrax analysis in 56.7% of the 384 dogs tested. Prevalence was defined as the greatest number of same breed signatures identified with two or more great-grandparents. Broadening the criteria for agreement to ignore the order in which breeds were labeled by shelter staff (i.e., primary and secondary), we found that 67.7% of these dogs had at least one breed identified by staff that agreed with DNA analysis, while 33.3% of visual breed assignments by staff did not match any of the up to eight breeds indicated in the analysis. Staff only matched both the primary and secondary breeds in 10.4% of dogs (40 out of 384). We describe these as a “complete match.” In all but four of those cases, the breeds were in the same order; however, more than half of the complete matches by shelter staff (55.6%) concerned purebred dogs.

Of the 400 dogs at SDHS for whom both breed assignment and DNA testing results were obtained, 124 were identified as having a breed heritage consisting of at least one pit bull-type relative. Considering those dogs in whom the pit bull-type concentration was 25% or higher (114 dogs), shelter staff matched these dogs’ DNA analyses by identifying their primary breed assignment as a pit bull-type in 67.0% of cases. An additional 8.8% of dogs’ breed assignments by staff were in agreement when including assignments that were placed in the secondary breed position.

Twenty-seven dogs of pit bull-type heritage were not identified by shelter staff as pit bull-type and thus disagreed with DNA analysis. Of those 27 dogs, 20 (74.1%) were only one-quarter pit bull-type. Most commonly, mismatched dogs were listed as Labrador Retriever mixes by the staff. Conversely, four of the 270 dogs that did not have any pit bull heritage in their DNA analysis were identified as pit bull-type dogs by shelter personnel (Table 7). The DNA for these dogs showed them to be either Boxer or Rottweiler mixes.

**Table 7:** Confusion matrix for pit bull-type dog identification comparing dogs assigned a pit bull-type breed by staff and confirmation by DNA analysis for dogs with pit bull-type heritage of 25% or greater.

		*Assigned as pit bull by staff*		
		Yes	No	Total
*Confirmed pit bull* *by DNA analysis*	Yes	87	27	114
	No	4	266	270
	Total	91	293	384

**Fig 2:**
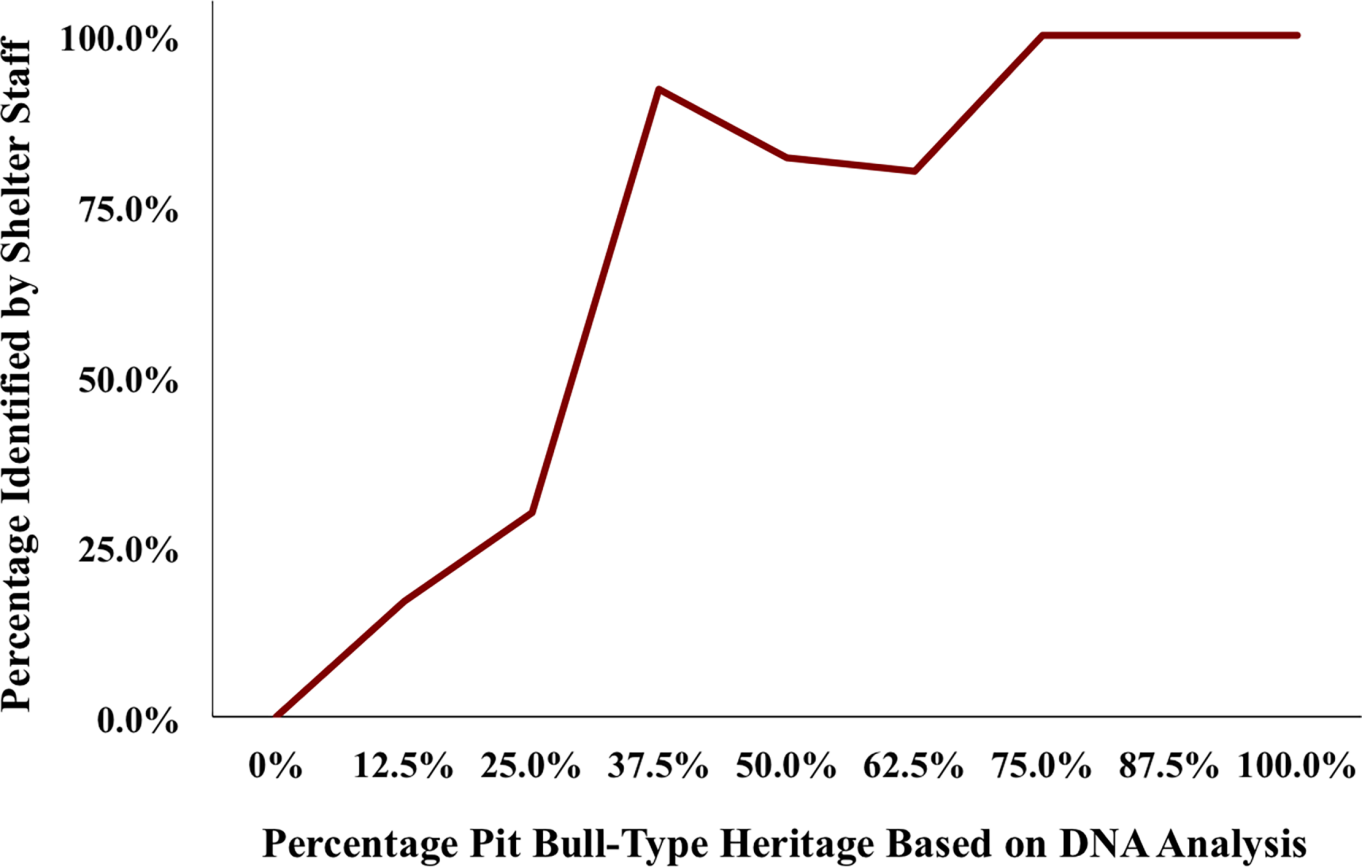
Percentage of pit bull-type dogs matched and mismatched by shelter staff by percentage of pit bull-type heritage.

In exploring the relationship between identification and pit bull heritage, we found a significant correlation between the number of DNA-identified pit bull-type relatives and the probability that shelter staff identified the dogs as pit bulls (*r* (85) = .75, *p* < .001). Dogs whose heritage was 25% pit bull or less were the most likely to be misidentified by staff as not having any of these breed ancestors. Conversely, shelter personnel were 92% successful in identifying dogs with 75% pit bull heritage or higher in their DNA analysis (Fig 2).

## Discussion

By testing over nine hundred dogs at two animal shelters in the United States, we were able to satisfy our aim of better understanding the breed identities of shelter dogs via commercially available genetic testing. To our knowledge, this is the largest reporting of breed heritage in sheltering to-date.

While organizations such as the Humane Society of the United States have reported that 25% of shelter dogs are purebreds [38], the results of our study do not confirm this number. Instead, we found approximately 5% of shelter dogs consisted of only one breed with the majority of these purebreds identified in San Diego.

The overall occurrence of purebreds in our sample is much lower than proportions of purebred dogs surrendered to shelters by their owners reported in Salman et al. [6] and Patronek, Glickman, and Moyer [5] (30 and 40 percent, respectively). There are several possible reasons for this discrepancy between our study and these earlier reports.

First, neither of the earlier reports used genetic breed testing: thus their estimates, based solely on owner report and visual identification, may be unreliable. Second, the reduced presence of stray dogs in our sample as compared to the national average [1] may have diluted the overall prevalence of purebreds. Third, another possibility for this discrepancy is that previous findings are roughly twenty years old, and shelter demographics have changed.

Kass, Johnson, and Weng [39] reported two California counties saw a reduction of over 35% in the number of dogs arriving at shelters from 1993 to 2006, and Morris and Giles [40] reported a reduction of nearly 45% in the number of dogs taken into Denver area shelters between 1998 and 2010. The latter study attributes years of low-cost spay and neuter policies as a possible cause of these declines. If the number of purebreds arriving at shelters has, in fact, declined or is rarer than once believed, and the prevalence of dogs with a multiple breed heritage is more common, it is possible then that the source of pet overpopulation in these communities is not the irresponsible breeding of purebred dogs but mixed breed dogs having unwanted litters. Additionally, we find that the most popular breeds registered with the American Kennel Club in these cities during this time include Bulldogs, French Bulldogs, and Labrador Retrievers in San Diego and Labrador and Golden Retrievers and German Shepherds in Phoenix (American Kennel Club, personal communication, December 29, 2017). While some of these breeds were present as purebreds or were found to contribute to the mixed breed heritage of dogs in our sample, others were not, further indicating that purebred dogs may not be as large as an influence on the shelter population as once believed.

When considering pedigree status as a factor in adoption success, Diesel et al. [41] found purebreds had shorter shelter lengths of stay than mixed breed dogs; and Siettou, Fraser, and Fraser [42] and Lepper, Kass, and Hart [3] concluded that purebreds had a 1.67 and 1.43, respectively, times greater likelihood of adoption compared to mixed breed dogs. While it is recognized that dog breeds are perceived differently [28,43], it is unclear whether potential adopters in shelters are recognizing purebreds by their appearance and choosing based on beliefs about breed, or are influenced by how the shelter has conveyed their pedigree status. Given the low prevalence of purebreds found in our sample compared to the percentages that are often cited, shelter indications of breed status may be a more influential aspect in adopter decision-making than previously considered.

Although purebreds were very infrequent in our sample, American Staffordshire Terriers, Labrador Retrievers, and Yorkshire Terriers were the most commonly observed breeds of purebred dogs (with five each). Without enforced registration, it is difficult to know the prevalence of different dog breeds in the United States, but these breeds of purebreds are among the most commonly treated breeds at a major chain of pet hospitals in the United States [44]. Labrador Retrievers also have the highest number of registrations with the American Kennel Club [45]. When we consider the most often identified breed signatures within the mixed breed dogs were American Staffordshire Terrier (or more broadly a pit bull-type breed) and Chihuahua (the number one Banfield-treated breed), it seems possible that the prevalence of these dogs in shelters, whether as purebreds or as mixes, is more likely related to their popularity amongst owners than abandonment due to breed-specific problems [3].

Most animal shelters describe dogs as either purebreds, single breed with mixed or with primary and secondary breeds listed. Previously, breed label has been shown to affect dogs’ length of stay [46], as well as which dogs are selected for transfer programs [47], and may even play a role in how frequently lost dogs are reclaimed by their owners [48]. However, our results here suggest that the method of description used in shelters does not accurately represent the breed heritage of three-quarters of dogs sampled. Instead most dogs were comprised on average, of three breeds, with some dogs having up to five different breed signatures identified at the great-grandparent level. While the removal of breed labels has been suggested to improve adoptions and length of stay [28], it may also be a low-cost strategy that acknowledges the inherent breed complexity of homeless dogs in animal shelters.

At both shelters studied here, pit bull-type dogs waited longer to be adopted. Previous studies have also found these dogs have longer lengths of stay [28,4]. Particularly at the San Diego shelter, we found a relationship between the number of pit bull relatives that were indicated in the dogs’ breed heritage and increased time spent in the shelter awaiting adoption. More pit bull-type relatives in a dog’s heritage also meant staff were more likely to identify the dogs as pit bull. Together, this may suggest that as a dog’s heritage becomes more predominantly pit bull, both adopters and shelter staff are able to perceive this in the dog’s appearance.

If animal shelters want to fully characterize the breed heritage of today’s shelter dogs, genetic testing is a more comprehensive method of description than visual identification. In our sample, we found no evidence of a relationship between the number of breeds identified in a dog’s DNA analysis and their length of stay at the shelter, suggesting that a dog’s attractiveness as perceived by potential adopters is not governed by purebred status alone. Instead, it is likely that shelter dog attractiveness as perceived by shelter visitors is more strongly affected by certain aspects of the dog’s appearance, such as coat color, length, and overall size [49]. Using DNA-derived breed heritage could help shelters better infer the influence of breed upon outcomes like adoption, length of stay, and even adopter satisfaction.

Beyond well-established breed-specific behaviors such as retrieving and pointing [50], evidence for breed influences on a wider range of behaviors is mixed [51]. In Serpell and Duffy [52], responses from 7,124 owners pet dogs from the 30 most popular breeds on the Canine Behavioral Assessment and Research Questionnaire (C-BARQ) yielded distinct behavioral profiles of the dogs on a range of temperament dimensions. Statistically significant differences between breeds were found in mean values on several traits. However, the authors emphasize that, “…individual variation in C-BARQ scores within breeds are often as great or greater than the differences between breeds, and this limits our ability to talk about breed-specific or breed-typical personality traits based on these kinds of measures.” A similar finding was reported by Svartberg [53] using direct behavioral testing with the Swedish Dog Mentality Assessment on 13,097 dogs from 31 breeds. He reported that temperamental traits varied between groups of dog breeds but also noted “…relatively large variations within-breeds…” Furthermore, in both studies, some of the significant effects of breed were due to a single or small number of breeds, which were outliers to the typical pattern of data.

To our knowledge, no experimental study to-date has demonstrated evidence of temperamental differences due to specific breed influences in dogs of mixed breed heritage. Research conducted on breed crosses by Scott and Fuller [54] found that while behaviors such as approach or handling by a stranger could be attributed to specific gene contributions by breed, more complex behaviors could not. In general, in species more commonly studied in behavioral genetics, complex behaviors are often highly polygenic traits influenced by multiple genes and environmental factors [55].

Given the evidence that difference in temperament as a function of breed between purebred dogs is small relative to the differences in temperament within breeds, and the genetic complexity of temperament traits, it’s difficult to imagine how knowledge of a mixed breed dog’s breed heritage could provide useful information about its typical behavior. The diversity of breeds and combinations detected within our study further complicates these predictions. Thus we suggest that instead of using breed-specific behaviors as a guide to informing adopters about mixed breed dogs, animal shelters should behaviorally assess the dogs in their care and communicate those observations about the individual dogs to potential adopters.

A recent study by Turcsán, Miklósi, and Kubinyi [56] investigated owner’s perceptions of their dog’s behavior, mixed breed dogs were found to differ from purebred dogs in perceived trainability, calmness, and the presence of behavioral problems. This may suggest that mixed breed dogs are a special case of dogs with behavior not consistent with those of purebreds; however it is possible that unaccounted for differences in rearing, socialization, and past experiences could explain these differences.

Visual identification by shelter staff at SDHS matched at least one breed in a dog’s heritage over two-thirds of the time. This contrasts with prior research in which shelter staff and dog care professionals were found to match the DNA analysis in only 30–35% of dogs tested [13,14]. We also found incidences of over- and under-identification of pit bull-type dogs to be much lower than previously reported [27]. It is possible that the size of the sample, method of sampling, population of dogs sampled, and skill of the staff contributed to such differences. Our findings do suggest, however, that visual breed identification is much more difficult when assigning both a primary and secondary breed to mixed breed dogs, with only 5% matching the results of DNA analysis. When considering the breed complexity indicated with these dogs, breed assignment by visual identification appears to be a much more complicated endeavor than previously imagined and a likely untenable process for shelters to carry out successfully.

Regardless of a dog’s breed heritage, morphology remains an influential factor in adoption decision-making [57,58]. Tracking the physical characteristics, rather than visually-identified breed, of shelter dogs, and the outcomes of these animals, would help us understand the relationship of morphology to adoption success and length of stay [49]. It may also prove useful in clarifying preferred morphology from perceived breed and help transfer programs meet the supply needs of shelters across the United States [47].

In describing the number of purebred dogs in our study, our sampling method may have been unintentionally biased by the use of two limited admission facilities instead of municipal shelters that may have more purebred dogs in their care. However, a recent web-based survey of shelter dog adoption profiles across the United States arrived at similar proportions of purebred dogs at 18 shelters [59]. As our study only included one shelter each in Arizona and California, it is inevitable that other areas of the country may identify other breeds not found in our sample with differing prevalence rates. While we used the most established, commercially available genetic breed testing service in our study to identify the breed heritage of these shelter dogs, dogs found to be mixed or unknown at the third generational level may be identifiable as additional breeds are sequenced and added to the Mars DNA database.

## Conclusions

We found that over 100 breed signatures were identified at each shelter in our genetic breed testing with over 91 breeds shared between sites. Breed ancestries ranged from having one to five unique breed signatures identified (7%). On average, purebreds represented less than 5% of dogs tested with individuals most often having three breed signatures identified within their genetic heritage. In order of prevalence at AAWL and SDHS, American Staffordshire Terrier, Chihuahua, and Poodle were the most common breeds identified. Concurrent with previous studies, dogs with pit bull-type ancestries were found to have longer lengths of stay than other dogs. While the shelter staff at SDHS was able to successfully identify based on appearance at least one breed in the dog’s genetic heritage nearly two-thirds of the time, their ability to identify more than one breed in the DNA analysis fell to roughly 10% with over half of those properly-identified dogs being purebreds. We did find, though, that as the number of pit bull-type relatives increased in a dog’s heritage, so did the staff’s ability to match its breed type. Overall when we consider the complexity in breed heritage of these shelter dogs coupled with the failure to identify multiple breeds based on morphology and the lack of any scientific basis to judging how these breed signatures interact within the individual dog, we believe shelters should instead focus their resources on communicating the morphology and behavior of the dogs in their care to best support matchmaking and adoption efforts.

## Supporting information

S1 Table Breed and varieties detected in the Mars Wisdom Panel 2.0 DogTrax. (PDF)

S1 Text American Kennel Club Most Popular Breeds in San Diego, CA and Phoenix, AZ Correspondence. (PDF)

S2 Text Wisdom Health Wisdom Panel Accuracy of Breed Identification Letter. (PDF)
