# Supplementary material for: A canine identity crisis: Genetic breed heritage testing of shelter dogs
Source: PLoS One. 2018 Aug 23;13(8):e0202633. doi: 10.1371/journal.pone.0202633 (PMC6107223; doi:10.1371/journal.pone.0202633)
Supplement: S1 Table — (PDF) [file pone.0202633.s001.pdf]

Table 1. Breeds and Varieties Detected in Wisdom Panel 2.0 DogTrax

---

Affenpinscher, Afghan Hound, Airedale Terrier, Akita, Alaskan Klee Kai, Alaskan Malamute, American Bulldog, American English Coonhound, American Eskimo Dog, American Foxhound, American Hairless Terrier, American Staffordshire Terrier, American Water Spaniel, Anatolian Shepherd Dog, Argentine Dogo, Australian Cattle Dog, Australian Kelpie, Australian Koolie, Australian Shepherd, Australian Terrier, Basenji, Bassett Hound, Beagle, Bearded Collie, Beauceron, Bedlington Terrier, Belgian Malinois, Belgian Sheepdog, Belgian Tervuren, Bergamasco, Berger Picard, Bernese Mountain Dog, Bichon Frise, Black and Tan Coonhound, Black Russian Terrier, Bloodhound, Bluetick Coonhound, Boerboel, Border Collie, Border Terrier, Borzoi, Boston Terrier, Bouvier des Flandres, Boxer, Boykin Spaniel, Briard, Brittany, Brussels Griffon, Bull Terrier (Miniature and Standard), Bulldog, Bullmastiff, Cairn Terrier, Canaan Dog, Cane Corso, Cardigan Welsh Corgi, Catahoula Leopard Dog, Cavalier King Charles Spaniel, Cesky Terrier, Chesapeake Bay Retriever, Chihuahua, Chinese Crested, Chinese Shar-Pei, Chinook, Chow Chow, Cirneco dell'Etna, Clumber Spaniel, Cocker Spaniel, Collie, Coton de Tulear, Curly-Coated Retriever, Dachshund (Standard and Miniature in Shorthair, Longhair, and Wirehair), Dalmatian, Dandie Dinmont Terrier, Doberman Pinscher, Dogue De Bordeaux, English Cocker Spaniel, English Foxhound, English Setter, English Springer Spaniel, English Toy Spaniel, Entlebucher Mountain Dog, Field Spaniel, Finnish Lapphund, Finnish Spitz, Flat-Coated Retriever, Fox Terrier (Smooth, Toy, and Wire), French Bulldog, German Pinscher, German Shepherd Dog, German Shorthaired Pointer, German Spitz, German Wirehaired Pointer, Glen of Imaal Terrier, Golden Retriever, Gordon Setter, Great Dane, Great Pyrenees, Greater Swiss Mountain Dog, Greyhound, Harrier, Havanese, Ibizan

Hound, Icelandic Sheepdog, Irish Red and White Setter, Irish Setter, Irish Terrier, Irish Water Spaniel, Irish Wolfhound, Italian Greyhound, Japanese Chin, Japanese Spitz (Klein and Mittel), Jindo, Keeshound, Kerry Blue Terrier, Komondor, Kuvasz, Labrador Retriever, Lagotto Romagnolo, Lakeland Terrier, Lancashire Heeler, Large Münsterlander, Leonberger, Lhasa Apso, Löwchen, Maltese, Manchester Terrier (Standard and Toy), Mastiff, Miniature Pinscher, Neapolitan Mastiff, Newfoundland, Norfolk Terrier, Norwegian Buhund, Norwegian Elkhound, Norwegian Lundehund, Norwich Terrier, Nova Scotia Duck Tolling Retriever, Old English Sheepdog, Otterhound, Papillon, Parson Russell Terrier, Pekingese, Pembroke Welsh Corgi, Petit Basset Griffon Vendeen, Pharaoh Hound, Plott Hound, Pointer, Polish Lowland Sheepdog, Pomeranian, Poodle (Toy, Miniature, and Standard), Portuguese Podengo Pequeno, Portuguese Water Dog, Pug, Puli, Pyrenean Shepherd, Rat Terrier, Redbone Coonhound, Rhodesian Ridgeback, Rottweiler, Russell Terrier, Saint Bernard, Saluki, Samoyed, Schipperke, Schnauzer (Giant, Standard, and Miniature), Scottish Deerhound, Scottish Terrier, Sealyham Terrier, Shetland Sheepdog, Shiba Inu, Shih Tzu, Siberian Husky, Silky Terrier, Skye Terrier, Small Münsterlander, Soft Coated Wheaten Terrier, Spanish Water Dog, Spinone Italiano, Staffordshire Bull Terrier, Sussex Spaniel, Swedish Vallhund, Tibetan Mastiff, Tibetan Spaniel, Tibetan Terrier, Treeing Walker Coonhound, Vizsla, Weimaraner, Welsh Springer Spaniel, Welsh Terrier, West Highland White Terrier, Whippet, White Swiss Shepherd, Wirehaired Pointing Griffon, Wirehaired Vizsla, Xoloitzcuintli, Yorkshire Terrier
