# Supplementary material for: A canine identity crisis: Genetic breed heritage testing of shelter dogs
Source: PLoS One. 2018 Aug 23;13(8):e0202633. doi: 10.1371/journal.pone.0202633 (PMC6107223; doi:10.1371/journal.pone.0202633)
Supplement: S1 Text — (PDF) [file pone.0202633.s002.pdf]

Hi Lisa,

Here are the most popular breeds for 2016:

San Diego

1. Bulldogs
2. French Bulldogs
3. Labrador Retrievers
4. German Shepherds/Golden Retrievers

6. Pembroke Welsh Corgis

7. Yorkshire Terriers

8. Siberian Huskies

9. Poodles

10. Dachshunds

Phoenix

1. Labrador Retrievers
2. Golden Retrievers
3. German Shepherds
4. Bulldogs
5. French Bulldogs
6. Cavalier King Charles Spaniels
7. Poodles
8. Rottweilers
9. Yorkshire Terriers
10. Australian Shepherds

I hope that helps!

Best,

-Bill

William Ellis

Communications Coordinator

American Kennel Club®
