# Supplementary material for: A canine identity crisis: Genetic breed heritage testing of shelter dogs
Source: PLoS One. 2018 Aug 23;13(8):e0202633. doi: 10.1371/journal.pone.0202633 (PMC6107223; doi:10.1371/journal.pone.0202633)
Supplement: S2 Text — (PDF) [file pone.0202633.s003.pdf]

WISDOM™ HEALTH

18101 SE SIXTH WAY
VANCOUVER, WA 98683
T: 360-944-2900
E: info@wisdomhealth.com

April 17, 2018

To whom it may concern,

I am writing to provide supporting documentation of the accuracy statement for breed identification in the WISDOM PANEL™ canine DNA tests performed as part of the manuscript submitted by Lisa Gunter.

The internal study undertaken to determine the accuracy of breed identification is described below.

Sample collection:

The WISDOM PANEL™ breed detection algorithm was tested using real mixed breed dogs. The vast majority of mixed breed dogs in the general population do not carry reliable records of breed history. Therefore, in order to undertake this phase of algorithm validation, simple known cross-bred animals were found. The primary source of such animals was the US commercial market for "designer" F1 crosses. These dogs are produced by crossing two different pure breeds to generate offspring with specific desired characteristics such as reduced coat shedding, miniaturization or other attributes of appearance. A wide variety of crosses are generated and, importantly for the purposes of this study, many carry reliable breeding records. However, despite the care that is commonly taken by breeders in documenting breed records, it is not a consistent practice to use exclusively AKC registered breeding stock. For enrolment into this study all dogs were required to carry reliable breed registration documentation. We also obtained samples from the colony dogs of two programs (a research program and a service dog breeding facility) which had well-documented ancestries for all individuals and where we were able to sample both parents and the F1 generation crosses.

In total, the test set of dogs with appropriate levels of registration comprised 246 individuals (predominately F1 dogs, but including a small number of F1 back-cross (F1B) and F2 dogs), thereby representing 492 parents. Independent statistical analysis powered the study to include a minimum of 204 F1 dogs.

Calculation of accuracy

To estimate performance metrics for the breed detection test the following rules were applied: test true positives defined as detection of any breed known to be present; false positive, detection of any breed known not to be present; false negative, not detecting any

breed known to be present. These values were then used to determine sensitivity and positive predictive value according to the following equations:

- Sensitivity = TP / (TP + FN)
- Positive predictive value = TP / (TP + FP)

Results

For the version of the WISDOM PANEL algorithm used for samples received from December 2014 to April 2016, the calculated Sensitivity and PPV values were greater than 90%. Both the Sensitivity and PPV calculations are independent of the number of breeds identified by the algorithm.

Best regards,

Angela Hughes DVM

Angela Hughes DVM PhD
Veterinary Genetics Research Manager
Wisdom Health
